# Disease perception, treatment-seeking behaviour and psychosocial impact of acne vulgaris among university students - A cross-sectional study

**DOI:** 10.51866/oa.622

**Published:** 2025-01-21

**Authors:** Yi Jun Tan, Adawiyah Jamil, Preamala Gunabalasingam

**Affiliations:** 1 MD, MRCP, Department Dermatology, Hospital Tuanku Ja’afar, Jalan Rasah, Bukit Rasah, Seremban, Negeri Sembilan, Malaysia. Email: tanyijun87@gmail.com; 2 MB BCh BAO, MMed, AdvMDerm, Department Medicine, Faculty of Medicine, Universiti Kebangsaan Malaysia, Jalan Yaacob Latif Kuala lumpur, Bandar Tun Razak, Cheras, Wilayah Persekutuan Kuala Lumpur, Malaysia.; 3 MBBS, MRCP, Adv M Derm, Department Dermatology, Hospital Tuanku Ja’afar, Jalan Rasah, Bukit Rasah, Seremban, Negeri Sembilan, Malaysia.

**Keywords:** Acne vulgaris, Perception, treatment-seeking behaviour, Psychology

## Abstract

**Introduction:**

Acne is a common chronic inflammatory disease. Misconceptions hinder effective management. This study aimed to explore disease perception, treatment-seeking behaviour and the psychosocial impact of acne.

**Methods:**

A cross-sectional study was conducted in four universities. A self-administered questionnaire was developed and validated. During clinical examination, acne severity was determined using the Comprehensive Acne Severity Scale (CASS) and psychosocial impact using the Cardiff Acne Disability Index (CADI).

**Results:**

Four hundred students with acne aged 20±1.62 years participated, among whom 62.5% were women. The self-perceived acne severity matched the CASS score in 54.4% of the participants but was worse in 37.5%. Approximately 80.5% correctly recognised acne as a disease, while beliefs about its chronicity varied. The aggravating factors were food (92.8%), genetic predisposition (92.8%), stress (91.3%), hygiene (86.3%) and menstruation (84.8%). The information sources were families (79.7%), online social media platforms (60.2%) and friends (58.5%). Doctor consultation was significantly associated with correct disease perception, severe disease and higher psychosocial impact. Cost was the commonest deterrent for seeking (63.8%) and discontinuing treatment (43.2%). The psychosocial impact was predominantly mild (71%). The CADI domains mostly affected were feelings and psychological state. The clinical (odd ratio [OR] =2.29, 95% confidence interval [CI] = 1.45, 3.61) and self-perceived acne severity (OR=4.83, 95% CI=2.79, 8.35) predicted a higher psychosocial impact.

**Conclusion:**

Misconceptions about acne as a disease were not prevalent, and aggravating factors other than food were correctly identified. Common information sources may further perpetuate misconceptions. Financial treatment barriers should be addressed especially in patients with severe acne and psychosocial impacts.

## Introduction

Acne is a chronic inflammatory skin disease affecting the pilosebaceous glands. It is clinically characterised by comedones, inflamed papules, pustules, nodules and cysts. The pathogenesis involves four primary factors: increased sebum secretion due to androgen stimulation, excessive follicular keratinisation, colonisation of follicles by Cutibacterium acnes and subsequent inflammatory reactions.

Epidemiological studies rank acne as the eighth most prevalent disease globally. 1 Contrary to common beliefs associating acne solely with adolescents, acne persists or appears beyond teenage years in many individuals. Its prevalence among young adults is rising, reaching as high as 40%-70%.^1,2^ Acne leads to complications such as scars, keloids and impaired psychosocial well-being. The level of social impairment increases with the severity of acne, which may lead to depression and anxiety.^3^

Online social media platforms are highly accessible and are common sources of information, particularly among young adults. Consequently, various myths and beliefs about acne, encompassing its nature, causes and treatment approaches, influence health behaviours.^4,5^ Many individuals do not regard acne as a disease, which needs medical attention despite its psychosocial impact. The rate of dermatologist consultation is as low as 18%.^6,7^ Misconceptions, stigmatisation and embarrassment are some factors preventing young adults from seeking medical treatment.^8^

We aimed to determine the perception of students in tertiary institutions towards acne as a disease, as well as its causes, aggravating factors and self-perceived severity. Treatment-seeking behaviours and factors that influence the source of treatment were also determined. Additionally, we explored the relationship between the severity and psychosocial impact of acne. Understanding patients’ attitudes can empower physicians to manage patients with acne more effectively and dispel misconceptions that hinder effective treatment.

## Methods

This study adopted a cross-sectional design, involving four tertiary educational institutions in Negeri Sembilan, Malaysia. The study period spanned from July to October 2023. Students who had acne, agreed to undergo skin examination and were proficient in the Malay language were included. Individuals with endocrine disorders associated with acne (e.g. polycystic ovarian syndrome, Cushing’s syndrome, acromegaly, congenital adrenal hyperplasia and androgen-secreting tumours) or acne-associated syndromes (e.g. pyogenic arthritis, pyoderma gangrenosum and acne syndrome; pyoderma gangrenosum, acne and hidradenitis suppurativa syndrome; and pyogenic arthritis, acne, pyoderma gangrenosum and hidradenitis suppurativa syndrome) and patients with known psychiatric illnesses such as anxiety disorders or schizophrenia were excluded.

A questionnaire in Malay (Malaysia’s national language) was constructed by two consultant dermatologists and a dermatology fellow. The questionnaire items were created based on the findings of previous studies on disease perception and treatment-seeking behaviour among patients with acne.^6,9,10^ While most of the core items were retained, some were either modified or added to fit the local context and ensure cultural relevance. The questionnaire items were aligned with the objectives of the study to effectively capture the perceptions towards, behaviours related to and impacts of acne. The questionnaire consisted of four sections. The first section captured information on participants’ acne history, including the age of onset, family history of acne and selfassessed severity of acne (i.e. mild, moderate or severe). The second section assessed participants’ perceptions of acne with 23 questions on the causes, disease course and aggravating factors of acne, with responses categorised as ‘yes’ or ‘no’. The third section explored treatment-seeking behaviour with 10 questions on the sources of information on acne, sources of treatment, types of treatment, expenditure on acne treatment, reasons for not seeking treatment and reasons for discontinuing previous treatment. The final section of the questionnaire integrated the Cardiff Acne Disability Index (CADI), a five-item questionnaire designed to assess the psychosocial impact of acne.^11^ Approval to utilise the validated Malay version of the CADI was obtained from Cardiff University. The CADI measures the psychological and social impacts of acne over the past month, focusing on aspects such as feelings of aggression, frustration, social life interference, avoidance of public changing facilities and perception of current acne severity. The CADI score ranges from 0 to 15, with higher scores indicating greater levels of disability and psychosocial impact. The scores are categorised as follows: 0=not impaired, 1-5=mildly impaired, 6-10=moderately impaired and 11-15=severely impaired.

A five-member expert panel, all dermatologists with more than 5 years of experience, three from the Ministry of Health, one from a public university and one from the private sector, rated the questionnaire in terms of clarity, relevance and simplicity on a 4-point ordinal scale (1=not relevant, 2=somewhat relevant, 3=quite relevant and 4=highly relevant). The number of panel members who rated the items as 3 or 4 was divided by the total number of panel members to obtain the content validity index of each item (Item-Content Validity Index [I-CVI] ). Generally, an item is deemed appropriate if the I-CVI is higher than 80%. Herein, the I-CVI of all questions was >80%, with an average value of 99.7%. Face validation was conducted with 10 patients aged 18-25 years attending the dermatology clinic at Hospital Tuanku Jaafar in Seremban, Malaysia, among whom four were Chinese, three were Malay and two were Indian. All questions were retained, as the strength of agreement per question and for the overall questionnaire ranged from 90% to 100%. Subsequently, a pilot study was conducted with 57 patients with acne aged 18-25 years from various ethnicities attending the same dermatology clinic. The questionnaire yielded a Cronbach’s alpha coefficient exceeding 0.7. This outcome confirmed the validity of the questions for utilisation in this study.

The investigators, accompanied by a lecturer, briefed students about the study and obtained informed consent. A participant information sheet, an informed consent form, a demographic data sheet and the questionnaire were provided via a Google Form link. Participants underwent clinical skin examination on the same day. The research team consisted of dermatologists and a dermatology fellow proficient in recognising dermatologic conditions and evaluating acne severity using the Comprehensive Acne Severity Scale (CASS). The CASS is used to determine the severity of facial acne, evaluated on a scale from 0 to 5. A score of 0 indicates clear skin; 1, almost clear skin; 2, mild acne; 3, moderate acne; 4, severe acne; and 5, very severe acne. The team conducted skin examinations, ensured completion of the questionnaires and addressed any incomplete submissions and questions from participants. Clinical skin examination focused solely on the facial area. Participants diagnosed with moderate-to-severe acne vulgaris were provided with referral letters to a public dermatology clinic if they wished for further treatment.

The sample size was calculated using the singleproportion formula, with a significance level of 5%, precision of 5% and a prevalence of acne among Malaysian university students of 71%, as reported in a previous study.^5^ Given 20% of incomplete data, the adjusted sample size was 385.

Data were analysed using the Statistical Package for the Social Sciences (version 26.0, SPSS, Chicago, IL, USA). Categorical variables were expressed as frequencies and percentages and numerical variables as means with standard deviations or medians with interquartile ranges according to their normality distribution. The chi-square test was utilised to determine the association between the study variables. The significance level for all tests was set at a 95% confidence interval (CI). A P-value of <0.05 was considered statistically significant.

Ethical approval was obtained from the Ministry of Health Medical Research and Ethics Committee (NMRR ID-23-00729-CZF and RSCH ID-23-00538-WCY).

## Results

A total of 400 students were included. Their mean age was 20±1.62 years. The onset of acne was within the age range of 8-23 years, with a mean age of 14±3.36 years. The majority (78.5%) reported a family history of acne. The demographic data are summarised in Table 1.

**Table 1:** Participant characteristics, clinical acne severity and self-perceived acne severity.

Characteristics	N=400 n (%) or mean±SD
Age, year	20±1.62
**Sex**	
Male	150 (37.5)
Female	250 (62.5)
**Ethnicity**	
Malay	289 (72.3)
Chinese	90 (22.5)
Indian	21 (5.2)
Age of acne onset, year	14±3.36
**Family history of acne**	
Yes	314 (78.5)
No	36 (9.0)
Not sure	50 (12.5)
**Self-perceived acne severity**	
Mild	156 (39.0)
Moderate	178 (44.5)
Severe	59 (14.7)
Very severe	7 (1.8)
**CASS score**	
Mild	284 (71.0)
Moderate	99 (24.8)
Severe	17 (4.2)
Very severe	-
**Category of self-perceived acne severity compared with the CASS score**	
Self-perceived acne severity consistent with the CASS score	218 (54.5)
Self-perceived acne severity worse than the CASS score	150 (37.5)
Self-perceived acne severity better than the CASS score	32 (8.0)

CASS: Comprehensive Acne Severity Scale

### Severity of acne

The participants’ self-perceived acne severity and dermatologists’ grading using the CASS are shown in Table 1. About half of the participants’ self-perceived acne severity was consistent with the CASS score. However, 150 (37.5%) participants perceived it as worse, while 32 (8.0%) perceived it as better (Table 1). There was a significant association between the participants’ self-perceived acne severity and dermatologists’ grading (Fisher’s exact test value=82.77, P<0.001). However, sex and ethnicity were not significantly associated with the participants’ perception of acne severity (P=0.600 and 0.100, respectively).

### Perceptions of acne as a disease

The majority of the participants (n=322, 80.5%) correctly recognised acne as a skin disease, while 297 (74.8%) knew that it is not contagious. Most participants (n=327, 81.8%) understood that acne can persist for over a year, and an overwhelming 388 (97.0%) were aware of its treatability. An almost equal number of participants was divided on whether acne disappears with age, with 199 (49.8%) agreeing. A significant proportion (n=263, 65.8%) viewed acne as a medical problem, while an even larger proportion (n=349, 87.2%) perceived it as a cosmetic issue. The majority (n=366, 91.5%) considered acne normal for their age (Table 2).

**Table 2:** Participants’ perception of acne as a disease and factors causing or aggravating acne.

Parameters	Participants’ perception N=400 n (%)	
Acne as a disease	Agree	Disagree
Acne is a skin disease.	322 (80.5)	78 (19.5)
Acne is contagious.	103 (25.8)	297 (74.8)
Acne is a chronic disease.	327 (81.8)	73 (18.3)
Acne can be treated.	388 (97.0)	12 (3.1)
Acne will disappear with age.	199 (49.8)	201 (50.3)
Acne is a medical problem.	263 (65.8)	137 (34.3)
Acne is a cosmetic problem.	349 (87.3)	51 (12.8)
Acne is normal for their age.	366 (91.5)	34 (8.5)
**Factors causing or aggravating acne**		
Food	371 (92.8)	29 (7.2)
Genetics	371 (92.8)	29 (7.2)
Stress	365 (91.3)	35 (8.8)
Hygiene	345 (86.3)	55 (13.8)
Menstruation	339 (84.8)	61 (15.3)
Face mask	331 (82.8)	69 (17.2)
Cosmetics	326 (81.6)	74 (18.5)
Sweat	305 (76.3)	95 (23.8)
Obesity	243 (60.8)	157 (39.2)
Sun exposure	239 (59.8)	161 (40.3)
Smoking	213 (53.3)	187 (46.8)
**Types of food aggravating acne**		
Fried foods	357 (89.3)	43 (10.7)
Sweets	281 (70.3)	119 (29.7)
Peanuts	277 (69.3)	123 (30.7)
Dairy products	232 (58.0)	168 (42)
Shellfish	230 (57.6)	170 (42.5)
Spicy foods	185 (46.3)	215 (53.7)

### Beliefs about acne

The participants identified various causes or aggravating factors contributing to acne. The top five factors were genetic predisposition (n=296, 92.8%), dietary habits (n=371, 92.8%), stress (n=365, 91.3%), hygiene (n=345, 86.3%) and menstruation (n=339, 84.8%). The participants believed that certain foods might contribute to acne. Fried foods were widely perceived as a trigger (n=357, 89.3%), followed by sweets (n=281, 70.3%) and peanuts (n=277, 69.3%) (Table 2).

### Treatment-seeking behaviours

Only 118 (29.5%) participants sought treatment for acne, utilising various sources of information. The sources of treatment information were categorised into doctor and non-doctor sources. Non-doctor sources included family members (n=93, 79.7%), online social media platforms such as Facebook and Instagram where individuals other than doctors provide advice (n=70, 60.2%), beauticians (n=31, 26.3%), pharmacists (n=44, 38.1%) and homoeopathy practitioners (n=6, 5.1%). Doctor sources comprised dermatologists (n=24, 20.3%), general practitioners (n=14, 11.9%) and doctors in aesthetic clinics (n=8, 6.8%).

The participants’ perception of acne as a disease was significantly associated with consulting a doctor (P=0.016) and seeking medical advice (P=0.016). The factors associated with consulting doctors rather than other sources were the severity of acne (P=0.009) and the psychosocial impact measured using the CADI (P=0.004) (Table 3).

**Table 3:** Factors associated with the chosen sources of treatment.

Parameters	Students seeking treatment n=118		P-value
	Non-doctors n=78 n (%)	78 (19.5) Doctors n=40 n (%)	
**Sex**			
Male	28 (35.9)	12 (30.0)	0.520^a^
Female	50 (64.1)	28 (70.0)	
**Ethnicity**			
Malay	54 (69.2)	30 (75.0)	0.660^b^
Chinese	21 (27.0)	8 (20.0)	
Indian	3 (3.8)	2 (5.0)	
**Age of acne onset, year**			
<17	73 (93.6)	33 (82.5)	0.100^b^
>17	5 (6.4)	7 (17.5)	
**Self-perceived acne severity**			
Mild to moderate	72 (92.3)	22 (55.0)	<0.001^a^
Severe and very severe	6 (7.7)	18 (45.0)	
**CASS score**			
Mild to moderate	49 (62.8)	15 (37.5)	0.009^a^
Severe and very severe	29 (37.2)	25 (62.5)	
**CADI score**			
Mild	55 (70.5)	20 (50.0)	
Moderate	20 (25.6)	11 (27.5)	0.004^a^
Severe	3 (3.9)	9 (22.5)	
**Perception of acne as a disease**			
Correct	55 (70.5)	26 (65.0)	0.016^a^
Incorrect	23 (29.5)	14 (35.0)	
**Amount of pocket money**			
<RM 500	75 (96.2)	38 (95.0)	0.870^b^
>RM 500	3 (3.8)	2 (5.0)	

a Chi-square test

b Fisher’s exact test.

Non-doctors: Families, online resources, friends, beauticians and pharmacists.

Doctors: Dermatologists, general doctors and doctors in aesthetic clinics.

CASS: Comprehensive Acne Severity Scale

CADI: Cardiff Acne Disability Index

Table 4 shows the types of treatment used by the participants, including topical and oral medications taken and procedures undergone.

**Table 4:** Types of acne treatment used by the participants.

Types of treatment	Frequency N=400 n (%)
Topical medications, n=104	104 (26.0)
Facial wash	104 (88.1)
Toner	78 (66.1)
Moisturiser	85 (72.1)
Acne cream	86 (72.9)
Facial scrub	44 (37.3)
Sheet mask	61 (51.7)
Night cream	42 (35.6)
Serum	78 (66.1)
Cleansing oil	45 (38.1)
Oral medications, n=30	30 (7.5)
Antibiotics	27 (22.9)
Retinoids	14 (11.9)
Supplements	30 (25.4)
Hormonal pills	11 (9.3)
Procedures, n=28	28 (7.0)
Facial treatment	28 (23.7)
Extraction	24 (20.3)
Laser therapy	9 (7.6)
Chemical peel	6 (5.1)
Steroid injection	1 (0.8)
Blue light or red light	7 (5.9)

The participants discontinued previous acne treatments primarily due to cost (43.2%) and forgetfulness (26.3%). Skin irritation and inconvenient application frequency were concerns for 20% each, while 5.9% experienced whitish dyspigmentation.

### Reasons for not seeking treatment

A total of 282 (70.5%) participants did not seek treatment due to cost (n=180, 63.8%), a belief that acne would self-resolve (50.7%), uncertainties in finding treatment sources (50.0%) and busy schedule (48.6%). Difficulty in accessing treatments (45.4%) and the perception of acne as a non-issue (36.9%) were other barriers. Conversely, shyness was cited by 21.3% of the participants.

### Psychosocial impact of acne

The mean CADI score was 4.21±3.07. The majority (71.0%) had a mild psychosocial impact; 24.7% had a moderate psychosocial impact; and the remaining 4.2% had a high psychosocial impact. The CADI domains mostly affected were feelings, psychological state and avoidance of public changing facilities. A significantly higher psychological impact was observed among the female participants (P=0.042) and those who had severe self-perceived acne and CASS score (both P<0.001) (Table 5).

**Table 5:** Factors associated with the psychosocial impact of acne.

Parameters	CADI scores			P-value
	Mild n=284 n (%)	Moderate n=99 n (%)	Severe n=17 n (%)	
**Sex**				
Male	105 (37.0)	43 (43.0)	2 (12.0)	0.042^a^
Female	179 (63.0)	56 (57.0)	15 (88.0)	
**Ethnicity**				
Malay	206 (72.0)	70 (71.0)	13 (76.0)	
Chinese	61 (22.0)	27 (27.0)	2 (12.0)	0.183^b^
Indian	17 (6.0)	2 (2.0)	2 (12.0)	
**Self-perceived acne severity**				
Mild	257 (90.0)	72 (73.0)	5 (29.0)	<0.001^a^
Moderate to severe	27 (10.0)	27 (27.0)	12 (71.0)	
**CASS score**				
1-2	205 (72.0)	56 (57.0)	6 (35.0)	<0.001^a^
3-5	79 (28.0)	43 (43.0)	11 (65.0)	

a Chi-square test

b Fisher’s exact test.

CASS: Comprehensive Acne Severity Scale

CADI: Cardiff Acne Disability Index

Multiple logistic regression analysis showed that the participants with moderate and severe acne based on the CASS scores had an adjusted OR of 2.29 (95% CI=1.45, 3.61) for a higher psychosocial impact after taking into account confounding factors such as age, ethnicity and sex. Conversely, the participants who had moderate and severe self-perceived acne had an adjusted OR of 4.83 (95% CI=2.79, 8.35) for a higher psychosocial impact.

## Discussion

Acne myths and misconceptions are common and have been documented^7,10^ in various study populations. Some of these misconceptions and beliefs are influenced by cultural and regional differences. The established risk and aggravating factors of acne include family history, high-glycaemic diet, dairy product consumption, stress and hormonal changes. Our study participants correctly listed genetic predisposition, dietary habits, stress and menstruation as causes of acne. Misconceptions related to hygiene were substantially common. Acne has been attributed to dirt,^3,7^ poor hygiene,^5,8^ insufficient facial cleansing^5,12^ and pollution.^3,5,10,12^ Studies from Asia consistently report hygiene as a perceived cause or aggravating factor of acne.^7,10,12^

Dairy products and high-glycaemic index foods are linked to acne due to the impact of insulinlike growth factors on sebum production.^13^ The foods commonly associated with acne among our study participants were fried foods, sweets and peanuts. Su et al. reported that spicy and fried foods were blamed for acne in their study involving 429 Singaporean students in tertiary institutions. 10 In Thailand, dairy, chocolates and sweets were thought to be the culprit by 19% of patients, while a previous local study reported spicy foods and peanuts.^7,12^ The effect of fried foods on acne is largely unknown. In their study on 714 adolescents in Kuwait, AlKhabbaz et al. did not show any significant association.^14^ Nuts were not associated with acne in two case-controlled studies.^15^ However, in Korea,^16^ nut intake was significantly higher among patients with acne. About 90% of our study participants thought that stress causes or aggravates acne. Although stress is commonly perceived as a risk factor of acne^5,7^ and generally accepted as an aggravating factor, data on this factor are limited. Comprehension of these associations is crucial to dispel myths and misunderstandings among students regarding acne and stress.

Patients’ self-perceived acne severity and complaints frequently differ from physicians’ objective assessments.^5^ This discrepancy may underscore societal pressures within an imageconscious culture, emphasising the pursuit of ‘perfect’ skin. Similar to Su et al.,^10^ we observed a weak correlation between the self-perceived acne severity and CASS score. The severity was correctly self-categorised by approximately 54% of the participants, but more importantly, slightly more than a third felt that their acne was worse. Healthcare providers should be aware that this inconsistency is common and demonstrate empathy towards patients with acne-related concerns. Patients’ self-perceived acne severity should also be considered in formulating treatment plans.

In this study, families, friends, television and print media were the traditional primary sources of acne-related information. Online platforms have become prevalent sources in today’s digital era.^7,9^ Inaccurate online information may lead to widespread misunderstanding. Understanding the sources patients rely on allows dermatologists and primary care physicians to address potential misconceptions effectively. In our study, the participants frequently sought information from their families and social media. More than 60% relied on the internet, aligning with global trends.^7,9^ Patients should be advised on accurate web-based resources to enhance patient education regarding acne. The observed associations between the participants’ accurate perceptions of acne as a disease and their inclination to consult a doctor, alongside their ability to assess their own acne severity correctly, emphasise the importance of correct information. A person’s understanding of their condition positively influences healthcare-seeking behaviour.^17^

Among 29.5% of our cohort who sought treatment, more than half obtained treatment from sources other than physicians. Topical medications were the most common treatment of choice, with cleansers, acne creams, moisturisers, toners and serums being the most favoured, similar to previous reports.^18,19^

This study revealed several key reasons for avoidance in seeking treatment. High treatment costs emerged as the primary deterrent, along with a prevalent belief that acne would resolve without intervention. Other reasons were uncertainty in finding suitable treatment options, busy schedules, perception of acne as a trivial issue and shyness. Our findings are in line with data from other large-scale surveys,^18,20^ which highlight the need for interventions that address financial constraints, increase access to care, promote health education and dispel misconceptions.

The participants with more severe acne and those experiencing substantial psychosocial impacts demonstrated a greater inclination to seek medical attention.^18,20^ We found high cost, forgetfulness, multiple daily applications and skin irritation as factors contributing to the discontinuation of acne treatments. Several studies identified ineffectiveness and side effects from topical medications as reasons for discontinuing acne treatment.^18,19^ In a large-scale worldwide cohort study investigating treatment non-adherence among patients with acne, Dreno et al. reported that poor adherence was due to side effects in older patients and forgetfulness in younger patients.^20^ Recognising these factors enables doctors to counsel patients effectively by addressing potential side effects and implementing strategies to prevent treatment discontinuation.

The mean CADI score and the distribution of percentages within each CADI domain in our study are consistent with the findings of several related studies.^2,12^ A considerable proportion of our participants experienced emotional distress, aggression, frustration or embarrassment. The interplay between acne and psychosocial issues often leads to adverse emotional reactions, with impacts on quality of life and self-esteem, and even thoughts of suicide.^2,3,8,10-12^ The university students in our study represented young adults in a pivotal stage of identity and social development. They exhibited impacts of acne on personal and social aspects; 52% experienced negative effects, with 14.3% reporting moderate-to-severe implications. A greater impact was seen among the female participants and those with higher self-perceived acne severity and CASS score. Women often exhibit a higher level of cosmetic concern and a greater perception of appearance-related issues and tend to rely more on social relationships, which potentially contribute to this heightened impact.^6^ Young adults with acne are highly susceptible to psychological challenges. Early detection is important, and effective interventions should be instituted to deter further progression.

The study has limitations that merit consideration. Self-reported data introduce potential biases, including recall and social desirability biases. A cross-sectional study design also provides a snapshot rather than a dynamic view of individuals’ experiences, hindering the establishment of causal relationships or tracking of changes over time. The study participants may incorrectly identify the qualifications of individuals providing treatment information in online media sources and some aesthetic clinics. Future research addressing these limitations can enhance the applicability and validity of our findings.

In summary, our study provides insights into the multifaceted aspects of acne among university students in Malaysia. Notably, the weak correlation found between self-assessment and formal dermatological evaluation highlights the impact of societal pressures on the perception of acne severity. Correct perceptions of acne as a disease were associated with an increased likelihood of seeking medical help, emphasising the pivotal role of accurate information in healthcare-seeking behaviour. Financial constraints emerged as a major obstacle influencing both treatment-seeking behaviour and treatment discontinuation. Students should be made aware of treatments provided with minimal cost at government-funded clinics to address this problem. The psychosocial impact of acne, particularly among women, emphasises the need for early detection and effective interventions to mitigate adverse emotional reactions. Understanding the barriers to seeking treatment, including misconceptions and practical obstacles, is crucial for targeted healthcare interventions.

## Data sharing statement

No additional data beyond what is presented in this manuscript will be shared.

**How does this paper make a difference in general practice?**

- **Enhanced understanding:** The study offers insights into prevalent myths and accurate perceptions surrounding acne.
- **Informed healthcare practices:** It highlights the weak correlation between self-perception and clinical assessment, emphasising the importance of informed healthcare decisions.
- **Targeted patient education:** It identifies online platforms as influential information sources, emphasising the need for accurate web-based resources to enhance patient education regarding acne.
- **Improved treatment strategies:** It reveals treatment-seeking behaviours and barriers, enabling healthcare providers to tailor interventions, address financial constraints and dispel misconceptions.
- **Psychosocial impact recognition:** It recognises the psychosocial impact of acne, prompting early detection and tailored interventions for improved mental well-being.

## References

[1] Chen H, Zhang TC, Yin XL (2022). Magnitude and temporal trend of acne vulgaris burden in 204 countries and territories from 1990 to 2019: an analysis from the Global Burden of Disease Study 2019.. Br JDermatol..

[2] Lim TH, Badaruddin NSF, Foo SY (2022). Prevalence and psychosocial impact of acne vulgaris among high school and university students in Sarawak, Malaysia.. Med J Malaysia..

[3] Altunay IK, Ozkur E, Dalgard FJ (2020). Psychosocial aspects of adult acne: data from 13 European countries.. Acta Derm Venereol..

[4] Clatici VG, Satolli F, Tatu AL, Voicu C, Draganita AMV, Lotti T (2018). Butterfly effect - the concept and the implications in dermatology, acne, and rosacea.. Maedica (Bucur)..

[5] Markovic M, Soldatovic I, Bjekic M, Sipetic-Grujicic S (2019). Adolescents’ self perceived acne-related beliefs: from myth to science.. An Bras Dermatol..

[6] Savo I, Jorgaqi E, Vasili E, Mishtaku S, Demaj D, Jafferany M (2020). Treatment-seeking behavior, knowledge and beliefs about acne vulgaris among adolescents: a cross-sectional study in high school students in Tirana, Albania.. Dermatol Ther..

[7] Wisuthsarewong W, Nitiyarom R, Kanchanapenkul D, Arunkajohnask S, Limphoka P, Boonchai W (2020). Acne beliefs, treatment-seeking behaviors, information media usage, and impact on daily living activities of Thai acne patients.. J Cosmet Dermatol..

[8] Loh KC, Chan LC, Phang LF (2020). Perceptions and psychosocial judgement of patients with acne vulgaris.. Med J Malaysia..

[9] Szepietowski JC, Wolkenstein P, Veraldi S, Tennstedt D, Machovcova A, Delarue A (2018). Acne across Europe: an online survey on perceptions and management of acne.. J Eur Acad Dermatol Venereol..

[10] Su P, Chen Wee Aw D, Lee SH, Han Sim Toh MP (2015). Beliefs, perceptions and psychosocial impact of acne amongst Singaporean students in tertiary institutions.. J Dtsch Dermatol Ges..

[11] Abdelrazik YT, Ali FM, Salek MS, Finlay AY (2021). Clinical experience and psychometric properties of the Cardiff Acne Disability Index (CADI).. Br J Dermatol..

[12] Kwan JW Lee HL, Low DE, Tang JJ (2019). Perception and psychosocial impact of acne vulgaris among secondary school adolescents in Ipoh, Malaysia.. Malays J Dermatol..

[13] Dall’Oglio F, Nasca MR, Fiorentini F, Micali G (2021). Diet and acne: review of the evidence from 2009 to 2020.. Int J Dermatol..

[14] AlKhabbaz M, Al-Taiar A, Saeed M, Al-Sabah R, Albatineh AN (2020). Predictors of acne vulgaris among adolescents in Kuwait.. Med Princ Pract..

[15] Suppiah TSS, Sundram TKM, Tan ESS, Lee CK, Bustami NA, Tan CK (2018). Acne vulgaris and its association with dietary intake: a Malaysian perspective.. Asia Pac J Clin Nutr..

[16] Jung JY, Yoon MY, Min SU, Hong JS, Choi YS, Suh DH (2010). The influence of dietary patterns on acne vulgaris in Koreans.. Eur J Dermatol..

[17] Wan V, Selvakumar R, Zhang Q, Fleming P, Lynde C (2024). The Acne Education Project: an educational initiative to improve acne health literacy and promote help-seeking behavior in young adolescents.. Pediatr Dermatol..

[18] Perche P, Singh R, Feldman S (2022). Patient preferences for acne vulgaris treatment and barriers to care: a survey study.. J Drugs Dermatol..

[19] Lam Hoai XL, De Maertelaer V, Simonart T (2021). Real-world adherence to topical therapies in patients with moderate acne.. JAAD Int..

[20] Dreno B, Thiboutot D, Gollnick H (2010). Large-scale worldwide observational study of adherence with acne therapy.. Int J Dermatol..

